# Rhes, a striatal enriched protein, regulates post-translational small-ubiquitin-like-modifier (SUMO) modification of nuclear proteins and alters gene expression

**DOI:** 10.1007/s00018-024-05181-8

**Published:** 2024-04-08

**Authors:** Oscar Rivera, Manish Sharma, Sunayana Dagar, Neelam Shahani, Uri Nimrod Ramĺrez-Jarquĺn, Gogce Crynen, Pabalu Karunadharma, Francis McManus, Eric Bonneil, Thibault Pierre, Srinivasa Subramaniam

**Affiliations:** 1 Department of Neuroscience, The Herbert Wertheim UF Scripps Institute for Biomedical Innovation and Technology, Jupiter, FL 33458 USA; 2 Present Address: National Institute of Cardiology Ignacio Chávez, Department of Pharmacology, Mexico, USA; 3 Bioinformatics and Statistics Core, The Herbert Wertheim UF Scripps Institute for Biomedical Innovation and Technology, Jupiter, FL 33458 USA; 4 Genomic Core, The Herbert Wertheim UF Scripps Institute for Biomedical Innovation and Technology, Jupiter, FL 33458 USA; 5 Institute for Research in Immunology and Cancer, Université de Montréal, Montréal, Québec Canada; 6 Department of Chemistry, Université de Montréal, Montréal, Québec Canada; 7 Department of Biochemistry and Molecular Medicine, Université de Montréal, Montréal, Québec Canada; 8 The Skaggs Graduate School of Chemical and Biological Sciences, The Scripps Research Institute, La Jolla, CA 92037 USA; 9 Norman Fixel Institute for Neurological Diseases, 3009 SW Williston Rd, Gainesville, FL 32608 USA

**Keywords:** Medium spiny neuron, SUMO E3-ligase, Gene regulation, Brain, Differentiation, Morphology, Signaling, Perinuclear membrane

## Abstract

Rhes (Ras homolog enriched in the striatum), a multifunctional protein that regulates striatal functions associated with motor behaviors and neurological diseases, can shuttle from cell to cell via the formation of tunneling-like nanotubes (TNTs). However, the mechanisms by which Rhes mediates diverse functions remain unclear. Rhes is a small GTPase family member which contains a unique C-terminal Small Ubiquitin-like Modifier (SUMO) E3-like domain that promotes SUMO post-translational modification of proteins (SUMOylation) by promoting “cross-SUMOylation” of the SUMO enzyme SUMO E1 (Aos1/Uba2) and SUMO E2 ligase (Ubc-9). Nevertheless, the identity of the SUMO substrates of Rhes remains largely unknown. Here, by combining high throughput interactome and SUMO proteomics, we report that Rhes regulates the SUMOylation of nuclear proteins that are involved in the regulation of gene expression. Rhes increased the SUMOylation of histone deacetylase 1 (HDAC1) and histone 2B, while decreasing SUMOylation of heterogeneous nuclear ribonucleoprotein M (HNRNPM), protein polybromo-1 (PBRM1) and E3 SUMO-protein ligase (PIASy). We also found that Rhes itself is SUMOylated at 6 different lysine residues (K32, K110, K114, K120, K124, and K245). Furthermore, Rhes regulated the expression of genes involved in cellular morphogenesis and differentiation in the striatum, in a SUMO-dependent manner. Our findings thus provide evidence for a previously undescribed role for Rhes in regulating the SUMOylation of nuclear targets and in orchestrating striatal gene expression via SUMOylation.

**Supplementary Information:**

The online version contains supplementary material available at 10.1007/s00018-024-05181-8.

## Introduction

The mRNA of Rhes (Ras homolog enriched in the striatum) has significant expression in the striatum, a region that control motor skills, cognitive function, and emotion  [1–4]. The expression of Rhes mRNA is stimulated by thyroid hormones. Additionally, Rhes has the ability to suppress the cAMP/PKA pathway and N-type Ca^2+^ channels (Cav 2.2) [5–8]. We have found several new roles for Rhes in the striatum. Rhes, in conjunction with RAS guanyl nucleotide-releasing protein guanine nucleotide exchange factor (RasGRP1-GEF), has the ability to suppress amphetamine-induced hyperactivity by establishing a protein–protein interaction network known as the "Rhesactome." [8]. Rhes and RasGRP1 facilitate the development of L-DOPA-induced dyskinesia (LID) through the involvement of mammalian target of rapamycin (mTOR) signaling in a pre-clinical model of Parkinson's disease (PD) [9, 10]. Rhes modulates autophagy through Beclin1 in a manner that is independent of mTOR [11]. We have also shown that Rhes has a crucial function in the striatal damage associated with Huntington's disease (HD). We found that Rhes interacts with mutant huntingtin, the genetic risk factor for HD, and enhances its solubility and striatal toxicity through modification by the post-translational small ubiquitin-like modifier (SUMO) [9, 12, 13].

In addition to PD and HD, Rhes is associated with tauopathy and mental disorders. Rhes has a crucial function in developing mutant tau-induced pathology through SUMO modification [14, 15]. Single nucleotide polymorphism (SNP) of the Rhes gene, *RASD2,* s were found associated with schizophreniapatients [16, 17], and a rare and conserved somatic mutation (R57H) was found in the *RASD2* in twins diagnosed with autism [18]. However, the molecular process through which Rhes participate in diverse brain disorders remains unclear. Our recent discovery that Rhes promotes the formation of tubular or tunneling nanotube (TNT) moves from cell-to-cell via TNTs [19] strongly indicates an involvement of Rhes in cell–cell communication functions in the brain [20].

TNTs are delicate and inconspicuous membranous structures that connect two cells. TNTs have a diameter ranging from 50–200 nm and a length of 5–125 µm and have been observed in several types of cells [21]. Using mutagenesis, we have found that the membrane-binding site of Rhes (C263) is critical for the formation for TNTs. Furthermore, a distinct role for the GTPase domain (1-171aa) and the SUMO E3 ligase domain of Rhes (171-266aa) was identified in TNT formation [21]. The SUMO E3 ligase domain is necessary for TNT formation but is not on its own sufficient for the cell-to-cell transport. By contrast, the GTPase domain is defective in both the formation of TNT and in cell-to-cell transport [16].

Overall, the current evidence indicates that Rhes can execute more than one function at the molecular, cellular, and organismal levels. Multifunctional proteins like Rhes are not very rare in biology, as network topological information with protein annotations indicate that approximately 430 human proteins, including tumor suppressor protein p53 and cytochrome C, can be classed as extreme multifunctional proteins [22–24]. Despite the multiple roles and molecular complexity of Rhes, there is a lack of fundamental knowledge about the mechanisms by which Rhes operates. We previously  reported that Rhes physiologically regulates SUMO modification in vivo and mechanistically promotes the transfer of SUMO from thioester to lysine residues (“cross-SUMOylation”) between E1 (Aos1) and E2 (Ubc9) SUMO enzymes [25]. However, the SUMO substrates of Rhes remain unidentified. In the present study, we have used biochemical, proteomics, and RNA- seq tools to investigate the SUMO substrates of Rhes and the putative role of Rhes in striatal gene expression.

## Materials and methods

### Reagents, plasmids and antibodies

Unless otherwise noted, reagents were obtained from Sigma. Full length Rhes rat and human cDNA constructs, Myc, GST or GFP tagged, were cloned in pCMV vectors (Clontech) [12, 19, 26]. Mass spectrometry detection compatible His6-mSUMO1, His6-mSUMO2 and His6-mSUMO3 were cloned as described [27, 28]. p181 pK7-HDAC1 (GFP) (Addgene; #11054) were from Ramesh Shivdasani. H2B-mCherry (Addgene; #20972) were from Robert Benezra. mH2A1.2-CT-GFP (Addgene, #45169) were from Brian Chadwick, Hunt Willard. pT7-V5-SBP-C1-HshnRNPM (Addgene; #64924) were from Elisa Izaurralde. GFP-PBRM1 (Addgene; #65387) were from Kyle Miller. Flag-hPIASy (Addgene; #15208) were from Ke Shuai. GFP-NPM WT (Addgene; 15578)  were from Xin Wang. DGK beta (Addgene; #35405)  were from Robert Lefkowitz and Stephen Prescott. pCMV-L26-Flag (Addgene; #19972)  were from Moshe Oren. 6x-His Tag Antibody (clone HIS.H8) (1:1000, # MA1-21315) was from ThermoFisher Scientific. Antibodies for GST-HRP (1:5000, #sc-138), and Myc (1:3000, #sc-40) were obtained from Santa Cruz Biotechnology. mCherry antibody (1:1000, NBP2-25157)  was from Novus Biologicals. Flag antibody (1:1000, F7425) was obtained from Sigma-Aldrich. HA.11 Epitope Tag Antibody (1:1000, #901513) was from BioLegend (previously Covance catalog #MMS-101R). V5-Tag (1:1000, #13,202), GFP (1:1000, #2956), mTOR (1:3000, #2983), Histone H3 (1:10,000, #4499), MEK1/2 (1:1000, #8727) and LDH (1:5000, #2012) were from Cell Signaling Technology, Inc. Rhes antibody (1:1000, RHES-101AP) was from Fabgennix.

### Ni–NTA denaturing pull down for western blotting

Ni–NTA pull down of His-mSUMO3 conjugates was performed as previously described [12, 29]. Briefly, HEK293 cells (expressing transfected His-SUMO mutant and indicated constructs) were pretreated with proteosome inhibitor, MG132 (25 µM, 4 h), rinsed in PBS, scraped from 10cm^2^ dishes, and centrifuged at 750 × g for 5 min. Since a small fraction of proteins are SUMOylated, MG132 was added to stabilize SUMOylated proteins by preventing their proteasome degradation [28, 30, 31]. Cell pellets were then directly lysed in pull-downown buffer [6 M Guanidine hydrochloride, 10 mM Tris, 100 mM sodium phosphate] and sonicated. After collecting 50ul of sample for quantification, 40 mM imidazole and 5mM  β-mercaptoethanol was added to the remaining lysates and remaining pull-down buffer. imidazole and β-mercaptoethanol interfere with BCA protein assay. Lysates were then clarified by centrifugation at 3,000 × g for 15 min. All subsequent wash steps were performed with 10 resin volumes of buffer followed by centrifugation at 800 × g for 2 min. Ni–NTA Agarose beads (#30,210; Qiagen) were pre-equilibrated by washing three times with pull-down buffer. After equilibration, beads were resuspended in pull-down buffer as a 50% slurry of beads to buffer. After protein quantification of cleared cell lysates, 1 mg of lysate was added to 40 µL of Ni–NTA TALON bead slurry to a total volume of 1 mL in microcentrifuge tubes. The beads were then incubated overnight at 4 °C mixing end over end. The following day, beads were centrifuged at 4000 RPMs for 2 minutes at 4 °C  in a tabletop centrifuge and washed in the following sequence: once in pull-down buffer, once in pH 8.0 urea buffer (8 M Urea, 10 mM Tris, 100 mM sodium phosphate, 0.1% Triton X-100, 20 mM imidazole, 5 mM β-ME, pH 8.0), and three additional times in pH 6.3 urea buffer (8 M Urea, 10 mM Tris, 100 mM sodium phosphate, 0.1% Triton-X-100, 20 mM imidazole, 5 mM β-ME, pH 6.3). Elution was performed using 25 µL of Elution Buffer (pH 8.0 urea buffer containing 200 mM imidazole, 4X NuPAGE LDS loading dye, 720 mM β-ME). Samples were then heated at 95 °C for 5 min and directly used for Western Blotting. Inputs were loaded as 1% of the total cell lysate. The SUMOylation was quantified by normalizing the intensity of SUMOylation bands to the respective unmodified bands using Image-J software.

### GFP-Rhes localization studies

Approximately 75,000 STHdh^Q7/Q7^ cells were seeded on 35 mm glass bottom dishes. After 24 h the cells were transfected with indicated plasmids. Cells were imaged live using a Zeiss 880 confocal microscope at 63X oil immersion Plan- apochromat objective (1.4 NA) as before (22).

### Purification of SUMOylated Rhes interacting proteins

Briefly, GST + mSUMO3, and GST-Rhes + mSUMO3 (5 μg each) were transfected in 10 cm dish HEK293 cells, and after 48 h, cells were lysed in lysis/binding buffer [50 mM tris (pH 8.0), 150 mM NaCl, 10% glycerol, and 1.0% NP-40] with a protease inhibitor cocktail (Roche) and phosphatase inhibitor II (Sigma). For GST-affinity experiments, protein lysates were pretreated with glutathione beads for 1 h, glutathione beads were added, and the lysates were then rotated overnight at 4°C. The beads were washed three times in binding buffer without a protease inhibitor cocktail. The resulting purified material was subjected to denaturing Ni–NTA purification step to enrich for the SUMOylated interacting partners. The Ni–NTA bound resin underwent a thorough washing with 50 mM ammonium bicarbonate. Subsequently, the proteins were digested using trypsin for a duration of 16 h at 37 °C. The mSUMO3-modified peptides were immunoprecipitated using a custom anti-NQTGG antibody that targets the tryptic remnant generated on the lysine side chain of SUMO, followed by mass spectrometry identification as described before [30, 32, 33].

### SUMO peptide enrichment in Rhes overexpressing cells

Identification of SUMO modification lysine sites were carried out as described before [30, 32, 33]. Briefly, HEK293 cells were transfected with myc + mSUMO3 or myc-Rhes + mSUMO3, followed by denaturating Ni–NTA pulldown as described above. The Ni–NTA resin was extensively washed with 50 mM ammonium bicarbonate and the proteins digested with trypsin directly on the Ni–NTA solid support for 16 h at 37 °C. The mSUMO3-modified peptides were immunoprecipitated with anti-NQTGG antibody, as described before [30, 32, 33].

### Mass spectrometry

Samples were analyzed on the Q-Exactive HF instrument (ThermoFisher Scientific) and raw files processed using MaxQuant and Perseus, as described previously [30, 32, 33]. Briefly, samples were analyzed by LC–MS/MS on a Proxeon EASY-nLC system connected to Q-Exactive HF mass spectrometer (Thermo Fisher Scientific). Samples were loaded on a reverse-phase pre-column (5 mm length, 360 μm i.d.) and separated on a reverse-phase analytical column (18 cm length, 150 μm i.d.) (Phenomenex). Both columns were manually packed in-house. Separations were performed at flow rate of 0.6 μL/min using a linear gradient of 5–30% aqueous acetonitrile (0.2% formic acid) over 106 min. MS survey scans were performed at a resolution of 70,000 at m/z 200 with a mass window of m/z 350–1,500, maximum injection time of 200 ms and an automatic gain control of 1e6. MS/MS scans were acquired using a data dependent acquisition approach with a Top12 method for the proteome or Top speed of 3 s for SUMO peptides. The precursor isolation window was set to 2 m/z with a HCD normalized collision energy of 25, and a resolution of 17,500 at m/z 200. Automatic gain control (AGC) target values for MS/MS scans were set to 5e3 with a maximum fill time of 3 s. A dynamic exclusion of the previously acquired precursor ions was set to 15 s.

### Generation of KR mutants of Rhes

EGFP hRASD2[NM_014310.4] was first used as the template to generate five lysine to arginine (K to R) mutations at positions 32, 110, 114, 120 and 245 (5KR). It was then subcloned into myc vector backbone using specific primers with Sal1 and Not1 restriction sites. Three additional  K to R mutations were added at position 174, 175 and 191 on myc-Rhes_5KR template using a standard site-directed mutagenesis protocol with the respective primers containing the desired mutations. The desired mutations were confirmed by sequencing using CMV forward primer.

### SUMO1/2/3 knockout in striatal cells

Striatal STHdh^Q7/Q7^ cells deleted for SUMO1, 2 and 3 using CRISPR/Cas9 SUMO gRNAs as described [19]. Mouse gRNA sequences consisting of three oligo pools are as follows: SUMO1 CRISPR/Cas9 plasmid (SC-423588) (GGAGGCAAAACCTTCAACTG; GAGTTCCAATGAATTCACTC; GCCCGGTACCTGGTCAGACA), SUMO2 CRISPR/Cas9 plasmid (SC-431342. CCTCACCTGCCGTTCACAAT, GCTCACCTTGGGTTTCTCGT, CTTGTTAGGGTTTGTCAATG) and SUMO3 CRISPR/Cas9 (SC-423045, TTCCCCAGGGCTTGTCAATG, ACACACCTGCCTCTCACAGT, CCGTCGCTGCGCAACCATGT). CRISPR/Cas9 control plasmid (SC-418922) consisted of scrambled sequences.

### Mice

For organelle seperation and RNA-seq experiments, we used Rhes KO (*Rhes*^*−/*−^) mice, and C57BL/6 J mice. Rhes KO mice were obtained from Alessandro Usiello and were backcrossed with C57BL/6 J mice at least 8 generations; homozygous Rhes KO were used for all the experiments [9, 34]. WT mice (C57BL/6) were obtained from Jackson Laboratory and maintained in our animal facility according to Institutional Animal Care and Use Committee (IACUC) at The UF Wertheim Scripps Research Institute. Mice were euthanized by cervical dislocation and striatal tissues dissected and rapidly frozen in liquid nitrogen.

### Nuclear and cytoplasmic separation from striatum

The striatum from C57BL/6 J and Rhes KO mice was fractionated using the nuclear/cytosol fractionation kit according to the manufacturer’s instructions with minor modifications (BioVision). Briefly, striatum from C57BL/6 J and Rhes KO mice was rapidly dissected out and homogenized in CEB (cytosolic extraction buffer)-A with DTT and protease inhibitor, and incubating for 10 min on ice prior to addition of CEB-B. The lysates were vortexed for 5 s and incubated on ice for 1 min. The lysates were then centrifuged at 4 °C for 5 min at 16,000 × g in a microcentrifuge. And the supernatants were kept as the cytoplasmic fraction. The nuclear pellet was resuspended in NEB (nuclear extraction buffer). And vortexed the lysates for 15 s. This step was repeated 5 times every 10 min. The nuclear pellet was centrifuged at 4 °C for 10 min at 16,000 × g in a microcentrifuge. And the supernatants were kept as the nuclear fraction. The protein concentration was determined in the cytoplasmic and nuclear fractions using the BCA Protein Assay Kit (Pierce, Rockford, IL, USA). Equivalent amounts of protein samples (50 µg) were resolved by SDS-PAGE followed by immunoblotting as described below.

### Western blotting

Equal amounts of protein (20–50 µg) were loaded and were separated by electrophoresis in NuPAGE 4 to 12% bis–tris Gel (Thermo Fisher Scientific), transferred to polyvinylidene difluoride membranes, and probed with the indicated antibodies. HRP-conjugated secondary antibodies (Jackson ImmunoResearch Inc.) were probed to detect bound primary IgG with a chemiluminescence imager (Alpha Innotech) using enhanced chemiluminescence from WesternBright Quantum (Advansta). Where indicated the membranes were stained for ponceau.

### qPCR validation of targets

Striatum of mice (WT or Rhes KO) lysed in Trizol reagent. 250 ng RNA was used to prepare cDNA using Takara primescript kit (Cat no. 6110A) using random hexamers. The qRT-PCR of genes was performed with SYBR green (Takara RR420A) reagents. Primers for all the genes were designed based on sequences available from the Harvard qPCR primer bank. The primer sequences are as follows:

*Gapdh* mouse (Forward primer) 5’ primer AGGTCGGTGTGAACGGATTTG

(Reverse primer) 3’ primer TGTAGACCATGTAGTTGAGGTCA

*Nrp1* mouse (Forward primer) 5’ primer GACAAATGTGGCGGGACCATA

(Reverse primer) 3’ primer TGGATTAGCCATTCACACTTCTC

*Bhlhe22* mouse (Forward primer) 5’ primer TGAACGACGCTCTGGATGAG

(Reverse primer) 3’ primer GGTTGAGGTAGGCGACTAAGC

*Slit2* mouse (Forward primer) 5’ primer GGCAGACACTGTCCCTATCG

(Reverse primer) 3’ primer GTGTTGCGGGGGATATTCCT

*Epha5* mouse (Forward primer) 5’ primer AAGGAACCCTGTGGCTATTGG

(Reverse primer) 3’ primer GCAAACATGCCCGTTTTAGAGAA

*Dcx* mouse (Forward primer) 5’ primer CATTTTGACGAACGAGACAAAGC

(Reverse primer) 3’ primer TGGAAGTCCATTCATCCGTGA

*Ext1* mouse (Forward primer) 5’ primer TGGAGGCGTGCAGTTTAGG

(Reverse primer) 3’ primer GAAGCGGGGCCAGAAATGA

*Egr2* mouse (Forward primer) 5’ primer GCCAAGGCCGTAGACAAAATC

(Reverse primer) 3’ primer CCACTCCGTTCATCTGGTCA

*Mef2c* mouse (Forward primer) 5’ primer ATCCCGATGCAGACGATTCAG

(Reverse primer) 3’ primer AACAGCACACAATCTTTGCCT

*Plxna1* mouse (Forward primer) 5’ primer GGGTGTGTGGATAGCCATCAG

(Reverse primer) 3’ primer GCCAACATATACCTCTCCTGTCT

*Met* mouse (Forward primer) 5’ primer GTGAACATGAAGTATCAGCTCCC

(Reverse primer) 3’ primer TGTAGTTTGTGGCTCCGAGAT

*Efna5* mouse (Forward primer) 5’ primer ACACGTCCAAAGGGTTCAAGA

(Reverse primer) 3’ primer GTACGGTGTCATTTGTTGGTCT

### Global RNA-seq from WT and Rhes KO striatum

WT and Rhes KO mouse striatum (1 male and 1 female pooled per sample, 3 biological replicates per group) were lysed in Trizol and RNA extracted using the miRNeasy kit from Qiagen (cat. # 217,004). RNA was DNase-treated on column. Total RNA (500 ng) was depleted of ribosomal RNA using probes provided by the NEBNext rRNA depletion module (Cat. #: E6310L, NEB), according to manufacturer recommendations. The library preparation from the rRNA-depleted RNA was conducted according to NEBNext Ultra II Directional RNA kit (Cat. # E7760, NEB) guidelines. Briefly, the rRNA-depleted RNA samples were chemically fragmented and random hexamer primed for reverse transcription. First and second strand cDNA was generated with dUTP incorporation to the second strand. The ds cDNA was end repaired and adenylated at their 3’ ends. A corresponding ‘T’ nucleotide on the hairpin loop adaptors was utilized to ligate to the ds cDNA. The loop contains a dUTP that is removed along with all other incorporated U’s in the second strand by treatment with USER enzyme (Uracil-specific excision reagent). The degradation of the second strand in this step preserves directional sequencing of the intact first-strand thus preserving strand information of the RNA. The adaptor ligated DNA was PCR amplified with barcoded Illumina-compatible primers to generate the final libraries. The final libraries were sequenced on the NextSeq 500 with paired end 40 bp read lengths. Raw sequencing reads were quality and adapter-trimmed using Trimmomatic and mapped to the mouse genome (mouse-UCSC: M.musculus-UCSC-mm10: downloaded March 22, 2016) using STAR version 2.5.2a aligner and gene abundance was estimated with HTSeq version 0.8.0. Differential gene expressions were assessed with DESeq2. DESeq2 provides the greatest power (90%) for protein-coding genes with n = 3, while the power for long non-coding RNA is marginally lower. With three biological replicates per condition, DESeq2 has sufficient power to identify significant genes. In addition, these samples had a greater sequencing depth, with an average of 32 million reads per sample and a mapping rate of 55%, resulting in at least 17 million reads mapped to genes. With three biological replicates per condition and a high sequencing depth, the statistical power to detect significance in our reported genes should be sufficient. In addition, we only report genes whose p-values were lower than the FDR-adjusted p-value and should be adequately controlled for false positives.

Normalized gene counts were averaged and log10 transformed for WT and Rhes KO samples and were plotted against each other where Rhes KO log10 mean values were on x-axis and WT log10 mean values were on y-axis. Differentially regulated genes were identified using padj < 0.05 cut off and up (higher in Rhes KO) and down (higher in WT) regulated genes were marked with green and red respectively. The graph was generated using JMP®, Version 13.2.1. SAS Institute Inc., Cary, NC. The Top Diseased and Functions were generated through the use of QIAGEN Ingenuity Pathway Analysis (QIAGEN IPA) [35].

### Bioinformatic analysis with MaxQuant

Peptide identification from the raw files are searched using MaxQuant (version 1.6.2.10) [36]. MS/MS spectra are searched against Uniprot/SwissProt database that include Isoforms (released on November 6, 2020). The first search peptide tolerance is set to 20 ppm, the main search to 4.5 ppm, and fragment ion tolerance to 7.5 ppm. The maximum allowed number of missed cleavages by trypsin is set to 3 with a maximum of 5 modifications per peptide. Carbamidomethylation of cysteine residues is set as a fixed modification, while methionine oxidation, asparagine and glutamine deamidation, phosphorylation (STY), lysine SUMO3 (NQTGG) and protein N-acetylation are set as variable modifications. The false discovery rate (FDR) for peptide and protein is set to 1%, and the minimum peptide length is set to 6. Additional MaxQuant search parameters are listed in supplementary Data file S6.

### Statistical analysis

Data were expressed as means ± SEM. All the experiments were performed at least in triplicate and repeated twice at minimum. Statistical analysis was performed using Student's *t* test or one-Way ANOVA followed by multiple comparison test (GraphPad Prism 7).

### Data availability

Sequencing data have been submitted to the Gene Expression Omnibus (GEO) data repository, under the accession number GSE150990.The mass spectrometry proteomics data have been deposited to the ProteomeXchange Consortium via the PRIDE partner repository with the dataset identifier PXD023394. The account details for PRIDE: Username: reviewer_pxd023394@ebi.ac.uk. Password: iM1zNSnE.

## Results

### Rhes interacts with SUMOylated proteins

**Fig. 1:**
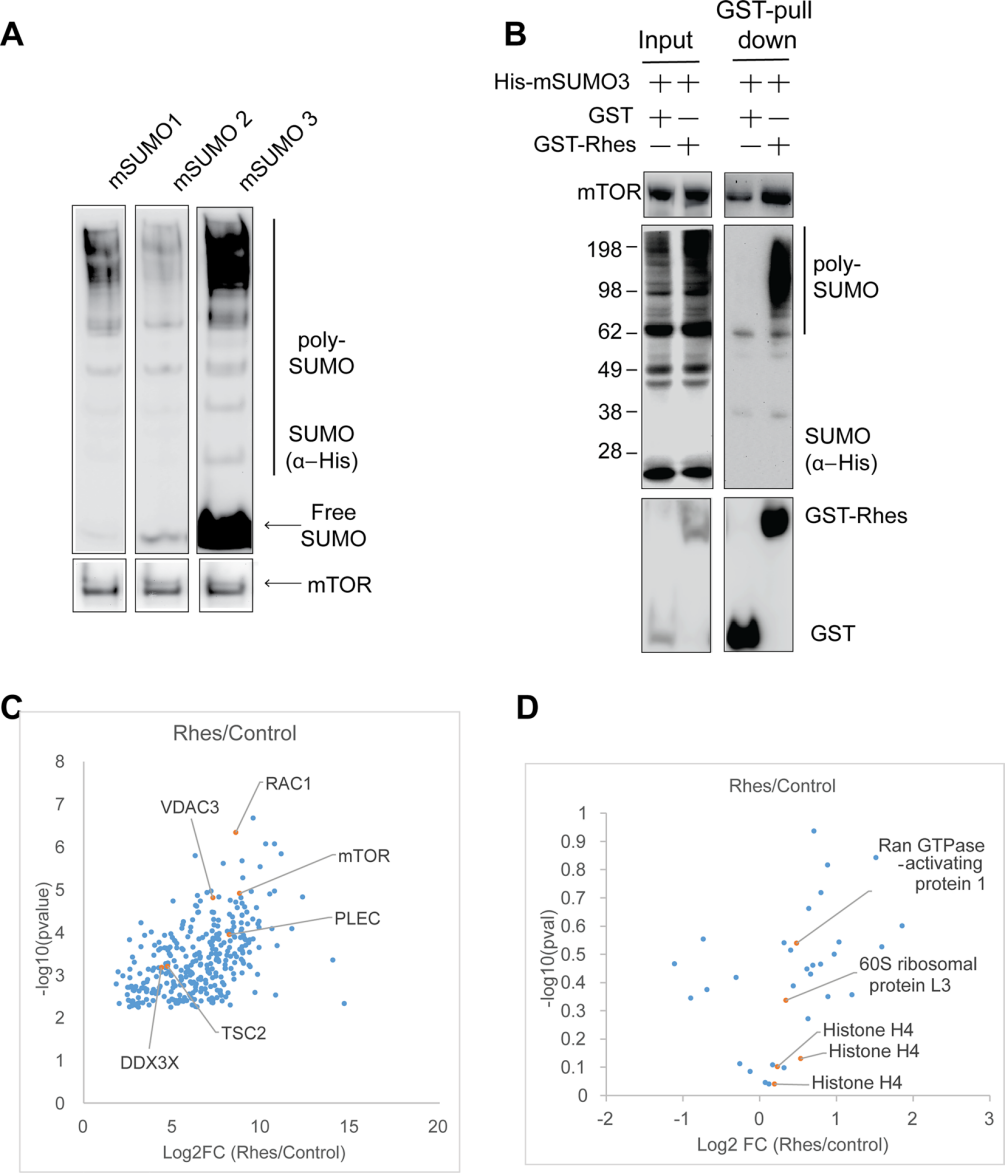
Rhes interacts with SUMOylated proteins. **A** Western blot of HEK293 cells expressing His-mSUMO1, His-mSUMO2, or His-mSUMO3. **B** Western blot for indicated proteins after glutathione-affinity pulldown in HEK293 cells expressing GST + His-mSUMO3 (control) or GST-Rhes + His-mSUMO3 and corresponding input (5%). **C** Volcano plot of proteins bound to affinity purified GST-Rhes (Rhes) co-expressing mSUMO3, compared to affinity purified GST co-expressing mSUMO3 (control), identified ~ 300 significant interactors by LC–MS/MS in biological triplicate. **D** Volcano plot of SUMOylated proteins that are bound to GST-Rhes (Rhes) + His-mSUMO3, compared to GST + His-mSUMO3 (control) identified by LC–MS/MS in biological triplicate. There was no substantial cut off in any of the proteins

Like ubiquitin, SUMO (which has 5 paralogues in vertebrates: SUMO1-5) is a conserved ~ 10.5 kDa protein modification that is covalently attached to lysine residues on multiple substrate proteins in a dynamic and reversible manner [37, 38]. We identified SUMO substrates of Rhes using an mSUMO3 construct in combination with a large-scale unbiased proteomics approach for SUMOylation site identification. The mSUMO3 structure has a mutation in the C-terminal that can be cleaved by trypsin, resulting in a C-terminus penta-peptide, which is compatible for mass-spectrometry (MS) detection [33, 39–41]. We employed mSUMO3 in this study because its transient expression produced more abundant protein than was produced by mSUMO1 or mSUMO2, suggesting that its use may aid in SUMO substrate detection in HEK293 cells (Fig. 1A).

First, we hypothesized that the SUMO proteins that bind to Rhes are the potential SUMO targets of Rhes. To examine this hypothesis, we co-expressed GST-Rhes or GST (control) with His-tagged mSUMO3 in HEK293 cells and affinity purified Rhes using a GST-glutathione-affinity column. We found that GST-Rhes readily bound to proteins that had been modified by mSUMO3 (Fig. 1B). The samples purified using the GST-Rhes affinity column was subjected to MS analysis to identify and quantify the interacting partners. We discovered several proteins that exhibit a strong and statistically significant binding affinity to Rhes, namely RAC1, TSC2, VDAC3, PLEC, DDX3X, and mTOR (Fig. 1C, Data file S1). These proteins have been previously recognized as interactors of Rhes [9, 10].

Attempts at identifying the interacting SUMOylated partners from the GST pulldown were futile. Although several targets, such as RPL3 (K399), histone H4 (K6, K9, and K13) and RANGAP1 (K524), were found SUMOylated, the log10-transformed p value for all of the targets all exceeded 0.05 (Fig. 1D, Data file S2). We reasoned that the SUMOylated proteins that are bound to Rhes are either very few in number or below the threshold needed for the detection by MS. Moreover, GST pulldown experiments are conducted under native conditions, which are also conditions under which the SUMO specific proteases (SENPs) are highly active and could deconjugate the SUMO moiety from the target proteins [42].

### Mass spectrometry reveals nuclear SUMO targets and SUMO modified lysines of Rhes

**Fig. 2:**
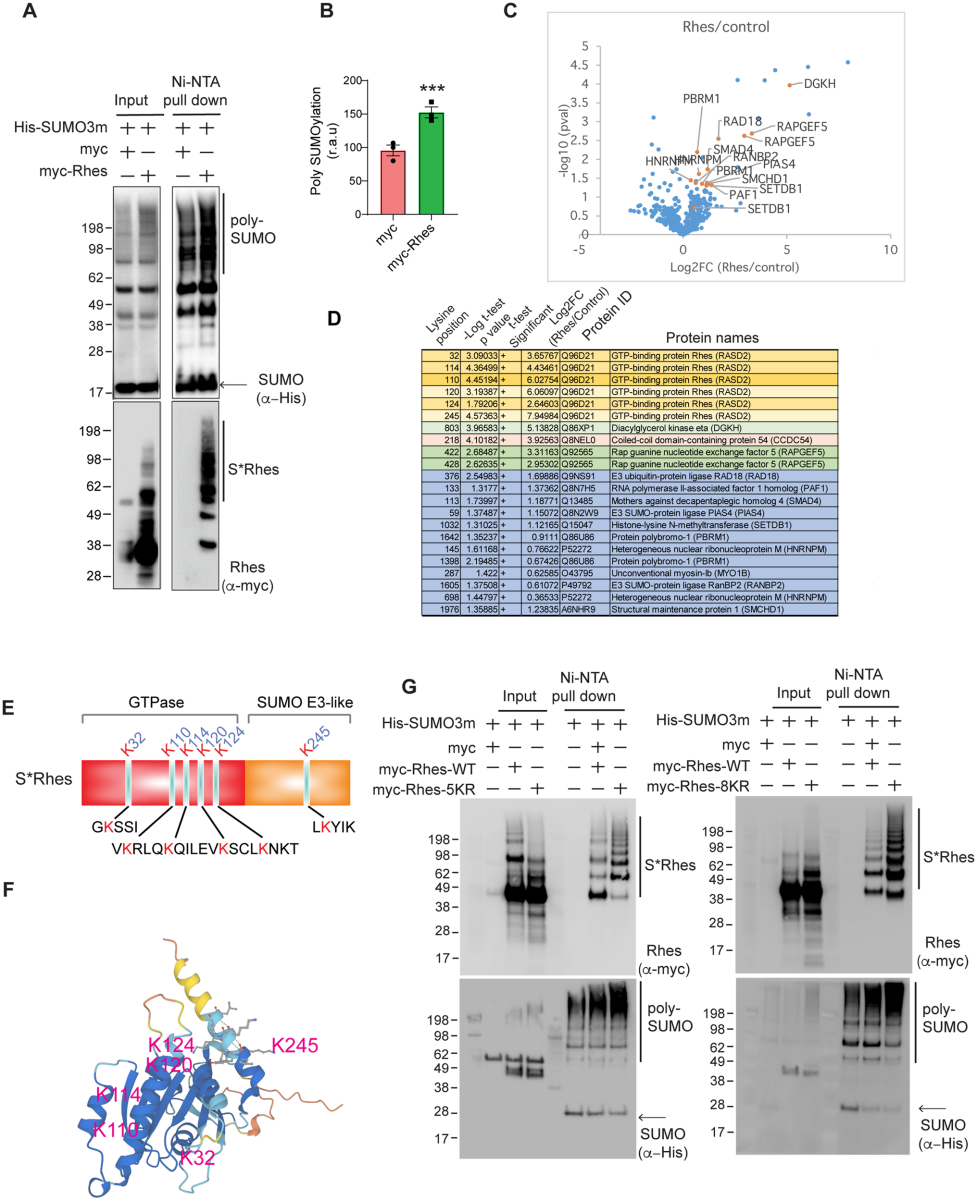
Interactome and SUMO proteome identifies putative SUMO substrates of Rhes. **A** Western blot of His-tagged SUMO enrichment using Ni–NTA TALON beads in HEK293 cells expressing myc + His- mSUMO3 or myc-Rhes + His- mSUMO3. S*Rhes represents SUMOylated Rhes. **B** Quantification of overall SUMOylation in Ni–NTA enriched, myc + His- mSUMO3 or myc-Rhes + His-mSUMO3. Data represents means ± SEM, (*n* = 3), ***P < 0.001, Student’s *t* test. **C** Volcano plot of SUMOylation site changes in myc-Rhes + His-mSUMO3 compared to myc + His-mSUMO3 (control), identified by LC–MS/MS in biological triplicate. **D** High and low confidence SUMO substrates of Rhes identified in *(C)* . **E** Representation of Rhes domains with mapping of the SUMO sites identified. **F** Structure prediction of Rhes from AlphaFold with MS-identified SUMO sites. **G** Western blot of His-tagged SUMO enrichment using Ni–NTA TALON beads in HEK293 cells expressing myc + His- mSUMO3 or myc-Rhes + His- mSUMO3, myc-Rhes 5KR + His- mSUMO3 or myc-Rhes 8KR + His- mSUMO3. S*Rhes represents SUMOylated Rhes

We then enriched the SUMOylated proteins using a denaturing purification protocol. We co-expressed myc-Rhes or myc (control) with His-tagged mSUMO3 in HEK293 cells and lysed the cells in guanidine-hydrochloride/urea denaturing buffer, followed by purification using a Ni–NTA column. We found that the myc-Rhes expression significantly enhanced the overall SUMOylation of proteins (Fig. 2A, B) and that Rhes was itself SUMOylated (S* Rhes, Fig. 2A), consistent with our previous report [12]. However, whether the increased SUMOylation seen in total lysates can be attributed to overexpressed Rhes SUMOylation remained unclear.

We identified the SUMO targets of Rhes by subjecting Ni–NTA-enriched myc and myc-Rhes samples (Fig. 2C) to SUMO peptide immunopurification using a custom antibody that recognizes the NQTGG remnant created on the modified lysine residue during trypsin digestion. Identification and quantification of the SUMOylation site by MS analysis identified several SUMOylated proteins showing a > fourfold change increase in SUMOylation, including 13 targets in myc-Rhes–expressing cells, compared to the myc control (Fig. 2D, Data file S3). We identified SUMO-regulated sites using a fold change in abundance > 4 between replicate analyses with p values < 0.05 (high confidence), while low confidence sites displayed p values < 0.05 and a fold change in abundance > 0 but < 4.

Among the high-confidence SUMO substrates, we found that Rhes increased the RapGEF5 SUMOylation at the lysine 422 and lysine 428 residues that are part of catalytic domain [40, 43]. RapGEF5 is a direct target of 3'-5'-cyclic adenosine monophosphate (cAMP) and is involved in cAMP-mediated signal transduction through activation of the Ras-like small GTPase RAPs: RAP1A, RAP1B, and RAP2A [41, 44]. We found that Rhes increased the SUMOylation at lysine 803 of diacylglycerol kinase eta (DGKH), an enzyme that generates phosphatidic acid (PA) and activates the Ras/B-Raf/C-Raf/MEK/ERK pathway. Rhes also increased the SUMOylation of CCDC54 (the coiled coil domain containing protein 54), a protein of unknown function, at lysine 218 (Fig. 2D).

Among the low-confidence SUMO targets, we found that Rhes promoted the SUMOylation of the SUMO-3 ligases PIASy (PIAS4) (K59) and RanBP2 (K1605), as well as the proteins polypbromo-1 (PBRM1, K1398, K1642), heterogenous nuclear ribonucleoprotein M (HNRNPM, K145, K698), histone-lysine N-methyltransferase (SETDB1, K1032), RNA polymerase II-associated factor 1 homolog (PAF1, K133), mothers against decapentaplegic homolog 4 (SMAD4, K113), and unconventional myosin 1b (K287) (Fig. 2D). Interestingly, Rhes was also one of the 4 high-confidence SUMOylated targets. Rhes was SUMOylated at six lysine residues (lys 32, 110, 114, 120, 124, 245 and 248) that spanned across the protein.

The MS/MS spectra of SUMOylated Rhes are presented in Supplementary Fig. 1. The GTPase domain of Rhes contains 5 SUMO modification sites, while the C-terminal SUMO E3-like region harbors a SUMOylation site (Fig. 2E); these sites are conserved across species (Supplementary Fig. 2). The Rhes AlphaFold structure also showed that K32, K120, and K124 are entrenched deeply in the structure's three-dimensional space, where they interact with other residues, whereas K110, K114, and K245 extend from the surface (Fig. 2F).

We then mutated these lysines to determine possible effects on the SUMOylation of Rhes. Surprisingly, the mutation of lysine at 32, 110, 114, 120, and 245 (5KR) or at 32, 110, 114, 120, 245, 174, 175, and 191 (8KR) did not diminish the SUMOylation of Rhes. Rhes contains up to 20 lysine residues (Supplementary Fig. 2), and the substitution of 8 of these lysines did not abolish SUMOylation, indicating that Rhes can also be modified by SUMOylation at multiple lysine residues. This phenomenon has also been observed for other SUMOylated proteins, such as H4, Cav-3, and α-synuclein [43–45].

Taken together, these data indicate that (a) Rhes modulates SUMOylation of substrates involved in cellular signaling, (b) Rhes is SUMOylated at multiple lysine residues and (c) the attachment of SUMO to a lysine residue is likely promiscuous within the Rhes protein.

### Comparison of the Rhes interactome and SUMOylation targets for further refinement of potential new SUMO targets of Rhes

While the quantitative SUMO proteome analysis (Fig. 2) identified new potential SUMO targets of Rhes, their abundance is limited. These results are not entirely unexpected because of the hurdles that are inherent in SUMOylation studies, such as the low abundance of the SUMO modified targets and the highly dynamic nature of SUMOylation. Indeed, at any given time, only < 1% of the total proteins are modified, further complicating their identification [46]. Finally, SUMOylation of certain substrates in native tissues, such as the striatum, may also be under the strict control of extracellular signaling.

**Fig. 3:**
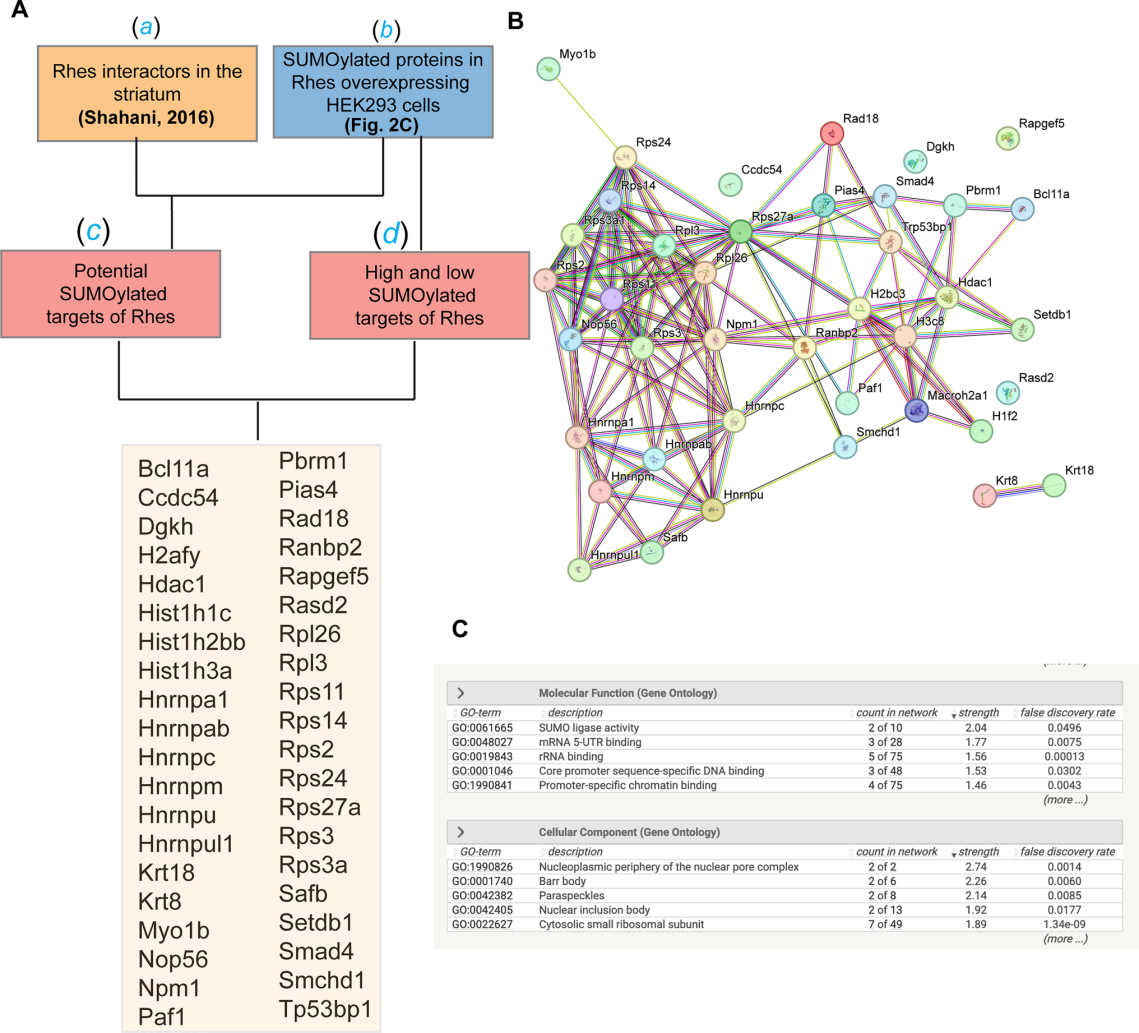
Flow chart to identify potential SUMOylated protein targets of Rhes. **A** Proteins that interacts with endogenous Rhes in striatum Shahani, 2016 [9] *(a)* are compared with all the SUMOylated proteins in HEK293 cells *(b)* and obtained 40 novel targets that are SUMOylated as well as binds to Rhes*.* **B** The STRING database analysis of protein–protein interactions of 40 potential targets of Rhes, and **C** Shows the significant evidence for molecular and cellular functions. Interaction score at STRING was set as medium confidence (0.400)

Keeping all these limitations in perspective, we sought to further identify the potential SUMO targets using an unbiased approach. Hypothesizing that the potential SUMO substrates of Rhes must interact with it, we selected the common proteins by comparing endogenous Rhes interactors in the mouse striatum [8] (Fig. 3A, (*a*)) with all the SUMOylated proteins in Rhes expressing HEK293 cells (Fig. 3C, (*b*)) (Data file S4). We have also included the high- and low-confidence SUMO substrates of Rhes in HEK293 cells (Fig. 2D). This cross-species protein comparison approach resulted in the identification of 40 novel substrates that are both SUMOylated and shown to interact with Rhes (Fig. 3B). The STRING analysis revealed strong protein–protein association networks and functional enrichment that included SUMO ligase activity, DNA binding activity, rRNA binding, 3’UTR binding, and translation initiation. Many of the targets, such as histone deacetylase 1 (HDAC1), PIASy, histone-2B, (H2B), HNRNPM, and PBRM1, have roles in gene regulation, indicating a potential nuclear function for Rhes.

### Rhes regulates SUMOylation of nuclear targets in cells

**Fig. 4:**
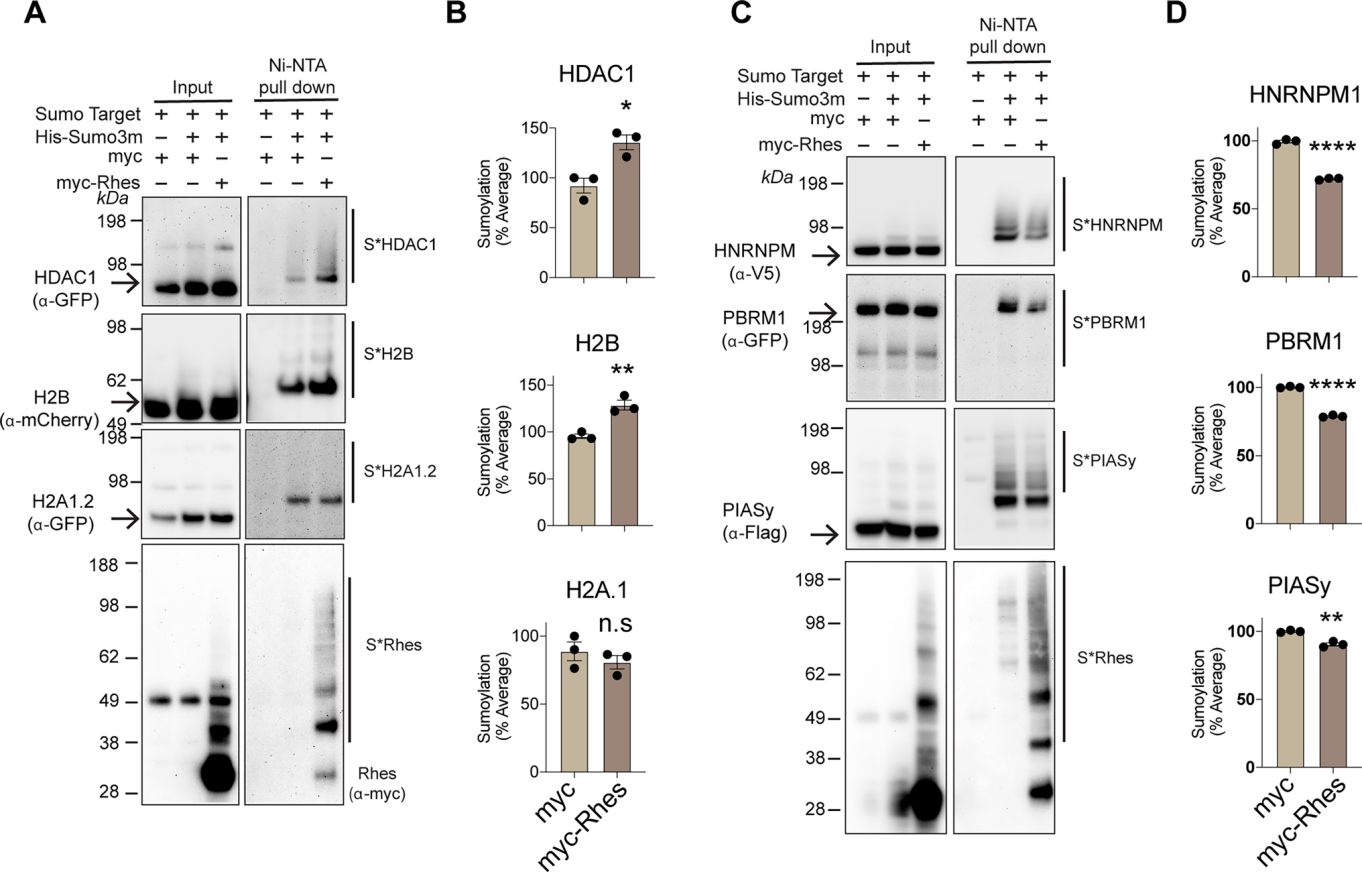
Rhes regulates the SUMOylation of nuclear targets. **A** Western blot of Ni–NTA enrichment of SUMOylated proteins (S*) from the lysates obtained from HEK293 cells expressing myc + His-mSUMO3 or myc-Rhes + His-mSUMO3 constructs either with GFP-HDAC1, m-cherry H2B or GFP-H2A1.2. **B**. Bar graphs indicate quantification of SUMOylation (%) from (A). Data represents mean ± SEM, (*n* = 3), *P < 0.05, **P < 0.01, Student’s *t* test. *n.s* not significant. (**C**) Western blot of Ni–NTA enrichment of SUMOylated proteins from the lysates obtained from HEK293 cells expressing myc + His-mSUMO3 or myc-Rhes + His-mSUMO3 constructs either with V5-HNRNPM1, GFP-PBRM1 or Flag-PIASy. **D** Bar graphs indicate quantification of SUMOylation (%) from (*C*). Data represents mean ± SEM, (*n* = 3), **P < 0.01, ****P < 0.0001, Student’s *t* test

We then investigated whether Rhes can directly modulate the SUMOylation of the nuclear targets identified in Fig. 4. We performed this using a Ni–NTA denaturing protocol, (Fig. 4) for the selected nuclear targets by expressing them in HEK293 cells.

We found that Rhes increased the SUMOylation of HDAC1 and H2B  but had no effect on the SUMOylation of histone H2A.1 (Fig. 4A, B). Surprisingly, Rhes decreased the overall SUMOylation of HNRNPM, PBRM1, and PIASy SUMOylation (Fig. 4B, C). We could not detect SUMOylation of other targets, including nucleophosmin (NPM), diacylglycerol kinase beta (DGKb), and ribosomal protein L26 (RPL26) (Supplementary Fig. 3). As expected, Rhes was readily SUMOylated under these experimental conditions (Fig. 4A–D). Taken together, these data indicated that Rhes differentially modulates the SUMOylation of substrates that are involved in nuclear functions, especially the regulation of gene expression.

### Rhes regulates the expression of genes involved in neuronal morphogenesis in the striatum

**Fig. 5:**
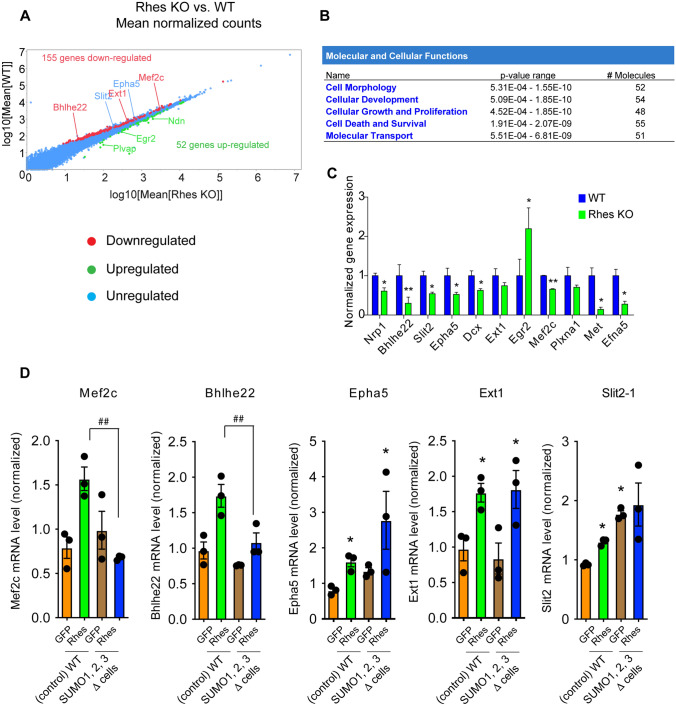
Rhes regulates genes involved in cellular differentiation and morphogenesis via SUMOylation. **A** Mean normalized counts of gene expression based on RNA seq data from Rhes KO (*Rhes*^−/−^) vs. WT (*Rhes*^+/+^) striatum. **B** Functional analysis of the molecular and cellular functions altered in striatum of Rhes KO mice compared to WT mice from (**A**) in Ingenuity Pathway Analysis. **C** Expression of indicated genes (normalized to *Gapdh*), involved in cell morphology and cellular development were validated by qPCR in Rhes KO vs. WT mice striatum. Error bar represents mean ± SEM, (*n* = 3), *P < 0.05, **p < 0.01 by Student *t* test. **D** Gene expression data for the indicated genes (normalized to *Gapdh*) in WT or SUMO 1, 2, 3 KO (Δ) cells to assess the effect of SUMOylation in presence of GFP or GFP-Rhes. Error bar represents mean ± SEM, (*n* = 3), *P < 0.05 compared to GFP in WT or SUMO 1, 2, 3 Δ cells ^##^*P* < 0.01 between Rhes in WT and Rhes in SUMO1,2,3 Δ cells by One-Way ANOVA followed by Newman-Keuls multiple comparison test

The finding that Rhes differentially regulates the SUMOylation of nuclear targets implicated in gene expression led us to hypothesize that Rhes may alter gene expression in the striatum in vivo. We addressed this possibility by isolating mRNA from WT and Rhes KO (*Rhes*^*–/–*^ ) mice (RNA from 1 male and 1 female mouse was pooled per sample, with triplicate samples prepared for each group WT or *Rhes*^−*/*−^) and conducting high-throughput RNA-seq analysis. We found that the absence of Rhes significantly altered the gene expression profile in the striatum (Fig. 5A). Of the ~ 15,000 sequenced genes, 155 genes were significantly downregulated and 52 genes were significantly upregulated in the *Rhes*^−*/*−^ mouse striatum compared to WT control mouse striatum (Fig. 5A, Data file S5). Ingenuity pathway analysis (IPA) showed the major hits to be the molecular and cellular functions involved in cell morphology, cellular development, growth and proliferation, and molecular transport (Fig. 5B). For example, genes involved in cell morphogenesis, including early growth response 2 (Egr2), necdin (Ndn), serum- and glucocorticoid-regulated kinase 1 (Sgk1), and netrin-1 (Ntn1), were upregulated [47–51], and neuropilin1 (Nrp1), basic-helix-loop helix 22 (Bhlhe22), and myocyte enhancer factor 2 (Mef2c) were downregulated [52–56] (Fig. 5A). Most genes, including ephrin type-A receptor 5 (Epha5) and slit guidance ligand 2 (Slit2), were unaffected. We also used qPCR for further validation of selected targets. We confirmed, for example, that Nrp1, Bhlhe22, Mef2c were downregulated, Egr2 was upregulated, and exostosin glycosyltransferase 1 (Ext1) showed a decreased trend) in the striatum of *Rhes*^*–/–*^ mice compared to WT striatum (Fig. 5C). These data indicate that Rhes regulates the expression of genes involved in a variety of biological processes and has a prominent role in determining cellular morphology in the striatum.

### Rhes regulates a selected gene expression via SUMO

Because Rhes regulates the SUMOylation of several proteins that are involved in gene expression such as HDAC1, HNRNPM, PBRM1 and PIASy [57–65], we investigated whether Rhes alters gene expression via SUMO. We compared CRISPR/Cas-9–control (WT cells) and CRISPR/Cas-9 SUMO1/2/3–depleted striatal neuronal cells that show ~ 60–70% of loss of SUMO (SUMO Δ 1, 2, 3 cells) [19]. We analyzed the effect of Rhes on the expression of* Ext1*, *Mef2*c, *Slit2*, *Epha5*, and *Bhlhe22* in WT and SUMO Δ 1, 2, 3 cells by transfecting the cells with GFP or GFP-Rhes and sorting using flow cytometry to obtain enriched population of cells expressing GFP or GFP-Rhes. While Rhes increased the expression of Mef2c and Bhlhe22 in WT cells, it failed to do so in the SUMO Δ cells (Fig. 5D). This result indicated that Rhes positively modulated the gene expression of Bhlhe22 and Mef2c via SUMO. Rhes increased Epha5 and Ext1 in both the control and the SUMO Δ 1, 2, 3 cells, indicating that Rhes promoted Epha5 and Ext1 expression through a mechanism that was independent of SUMO (Fig. 5D). Furthermore, while Slit2 was induced by Rhes in control cells, a high basal Slit2 expression was found in SUMO Δ cells that was not affected by Rhes expression (Fig. 5D). Collectively, these results indicate that Rhes regulates gene expression via SUMO-dependent and SUMO-independent mechanisms.

### Rhes is enriched in the perinuclear membrane fractions

**Fig. 6:**
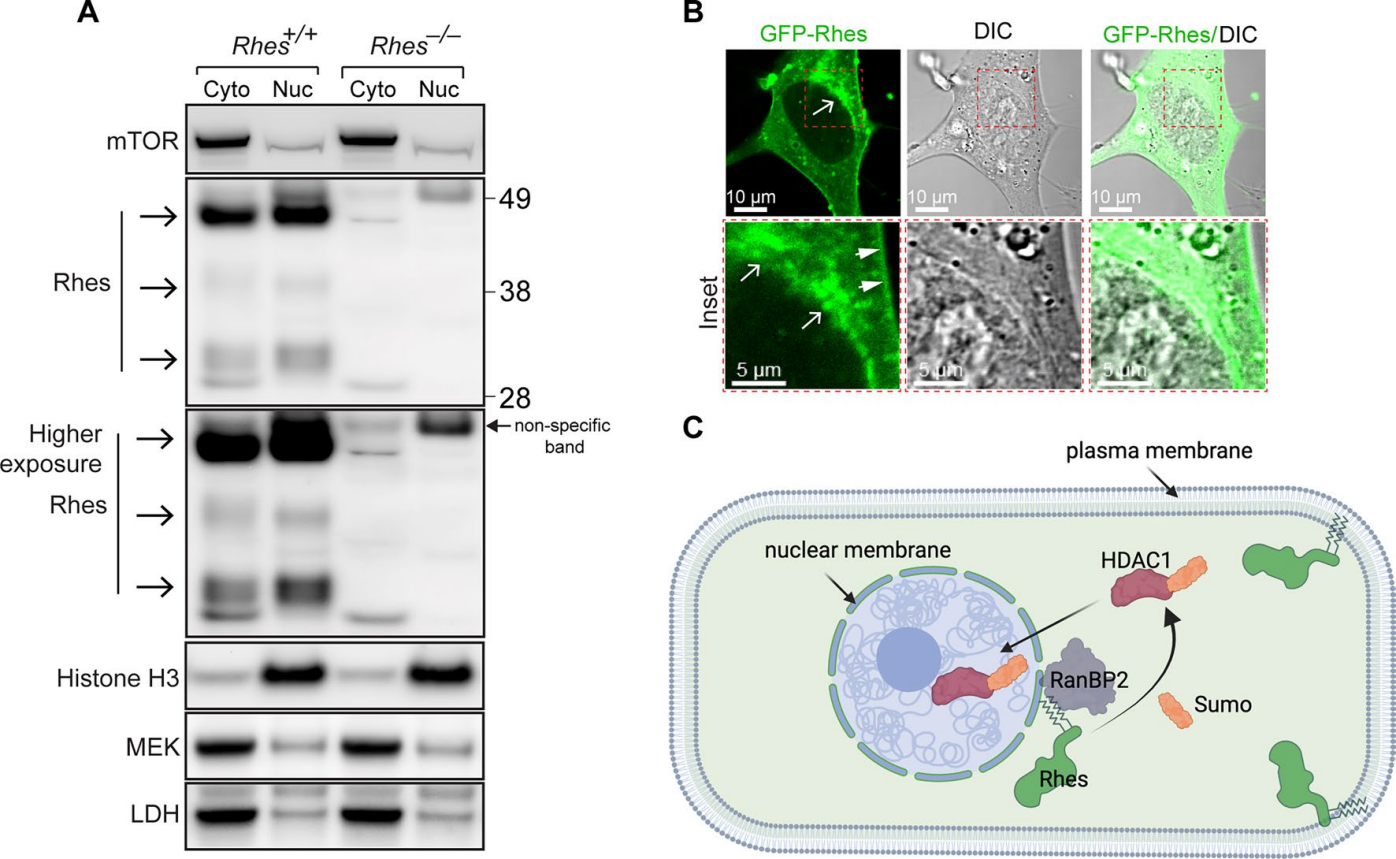
Rhes is preferentially localized around the perinuclear membrane. **A** Subcellular localization of Rhes was assessed in cytosolic and nuclear fractions from Rhes KO (*Rhes*^*−/*−^) vs. WT (*Rhes*^+*/*+^) mice striatum. The cytosolic markers MEK and LDH and the nuclear marker Histone H3 were probed to validate proper cellular fractionation. **B** Representative confocal and brightfield (DIC) image of striatal neuronal cell expressing GFP-Rhes, indicating the localization of Rhes on nuclear membrane and perinuclear space. Inset shows the boxed area at higher magnification. Arrow indicate perinuclear localization of Rhes, and arrowhead indicates the localization of Rhes on plasma membrane. **C** Model depicting that Rhes can SUMOylate its targets, including HDAC1, in the cytoplasm and promotes its entry into the nucleus to regulate gene expression

Our finding that Rhes mostly regulated the gene expression of selected nuclear targets prompted further investigation of whether Rhes is localized in the nucleus. Using biochemical tools, we isolated and separated cytoplasmic and nuclear fractions of the striatum from *Rhes*^+*/*+^ (WT) and *Rhes*^−*/*−^ (KO) mice (Fig. 6A). Using western blotting, we confirmed that Rhes was highly enriched in the nuclear fractions that were positive for histone H3 (Fig. 6A). Rhes was also observed in the cytoplasmic fractions that were enriched for the cytoplasmic markers mTOR, mitogen-activated protein kinase kinase (MEK), and lactate dehydrogenase (LDH) (Fig. 6A).

Because biochemically isolated subcellular fractions do not unmistakably report the distribution of subcellular proteins, we also verified the localization of Rhes using confocal fluorescence microscopy. Imaging analysis revealed that GFP-tagged-Rhes was predominantly associated with perinuclear membranes, with a negligible presence in the nucleus (Fig. 6B, arrows). In addition to its perinuclear localization, Rhes was also enriched in the plasma membrane (Fig. 6B, arrowhead) and in membranous vesicles, consistent with our previous reports [19, 63]. Taken together, our results indicate that Rhes is predominantly localized on the perinuclear membranes, indicating that Rhes may affect the SUMOylation of nuclear targets located in the perinuclear region (Fig. 6C).

## Discussion

The data presented here demonstrate the following: (a) Rhes is SUMOylated on multiple lysine sites; (b) Rhes regulates the SUMOylation of nuclear proteins, and (c) Rhes regulates gene expression in the striatum at least partly via SUMO-mediated mechanisms. Nevertheless, Rhes is well known to undergo post-translational modification in vivo [9, 64], although the nature of this modification remains unknown. This findings from the present study indicate that SUMO may contribute to this in vivo Rhes modification.

Interestingly, the presence of multiple closely spaced SUMO modification sites on Rhes at K110, K114, K120, and K124 indicates that these residues may act as anchors that can associate with SUMO-interacting motifs (SIMs), which have known involvements in protein–protein interactions [65, 66]. These closely spaced SUMOylation events are also found on other SUMO E3 ligases, such as the PIAS1 lysines K40, K46, K56, and K58; the PIASy lysines K59, K69, K128, K135; and the RanBP2 lysines K1596,d K1605, K2513, K2531, K2571, and K2592 [67, 68]. Thus, multiple lysine SUMO modifications appear to be a characteristic feature in many SUMO E3-like proteins. However, further study is needed to identify the role of each lysine modification and whether Rhes SUMOylates itself or if other potential SUMO E3 ligases SUMOylate Rhes. Other unanswered questions include, for instance, whether SUMOylation of Rhes regulates its activity toward mTORC1 kinase [10], TNT formation [19], or gene expression (Fig. 5). SUMOylation site mutants continue to attach to targets such as HDAC1 or H2b (Supplementary Fig. 4), indicating that alternative SUMO sites on Rhes may still connect to targets and presumably influence Rhes activity. These studies suggest that SUMOylation is flexible and can occur beyond a specified lysine, perhaps providing an evolutionary benefit for SUMO posttranslational modifications in molecular and cellular processes, while the exact cause of this flexibility is unknown.

Intriguingly, we report that Rhes differentially alters the SUMOylation of SUMO E3 ligases. Rhes decreased the overall SUMOylation on PIASy in the cells (Fig. 4B), while promoting the specific SUMOylation of PIASy on lysine 59 (Fig. 2D). Similarly, MS analysis revealed that Rhes significantly increased the SUMOylation of RanBP2 on lysine 1605, but not on lysine 2592 (Fig. 2D, Data file S3). These results indicate that Rhes may differentially affect the SUMOylation of SUMO E3 ligase(s) on specific lysine targets. An interesting future strategy will be to test whether PIASy or RanBP2 can act as a SUMO E3 ligase for Rhes. Even though SUMOylation is known to modify SUMO E3 ligases, the biological significance of the resulting modifications remains poorly understood. Previously, we reported that Rhes promoted cross-SUMOylation between the SUMO E1 (Aos/Uba9) and E2 (Ubc9) ligases and predicted that this regulation might work as a form of “symbiotic” regulation between two evolutionarily conserved SUMO E1 and E2 enzymes [25]. Based on the data presented here, we propose that Rhes may also promote reciprocal and symbiotic regulations between SUMO E3 ligases through SUMO modification and may therefore have a significant impact on the regulation of complex cellular and behavioral functions of the striatum via protein–protein interactions [9].

The results presented here clearly indicate that Rhes is involved in the regulation of gene expression in the striatum and that, intriguingly, it can both increase and decrease striatal gene expression. A previous independent study also reported that Rhes might inhibit gene expression by acting as a cis modulator [69], but the mechanisms were unclear. SUMOylation is a well-established participant in cis-regulation and is involved in both transcriptional repression and activation functions [46, 70, 71]. As Rhes KO cells show an overall diminishment of SUMOylation in the striatum [25], as well as altered gene expressions, we predict that the Rhes-SUMO pathway may regulate gene expression by functioning as a cis-modulator, possibly via the SUMOylation of transcription factors. Consistent with this notion, although our proteomics analysis did not reveal a significant alteration of HDAC1 SUMOylation on lysine 89 or lysine 476 (Data file S3), presumably due to low stoichiometry, our biochemical experiments showed that Rhes enhanced the SUMOylation of HDAC1 (Fig. 2B). Thus, Rhes may alter HDAC1 activity via SUMOylation at lysine 89 and at lysine 476, which is a catalytically essential residue involved in gene repression [72–74].

Previous studies have indicated an involvement of SUMOylation of H2B in the repression of gene expression [45]. Because we found that Rhes increases the SUMOylation of H2B (Fig. 3), which is SUMOylated at multiple lysine residues (Data file S4), including the previously reported lysine 6 [45], we propose that Rhes-SUMO pathway may affect gene expression via more than one nuclear target. However, the cellular compartment in which Rhes regulates the SUMOylation of nuclear proteins remains to be elucidated. Our finding that Rhes is enriched in the perinuclear space (Fig. 6B) leads us to predict that this perinuclear location may serve as the prime site for protein SUMOylation (Fig. 6C). In support of this notion, previous studies have shown that the SUMO E3 ligase RanBP2 is localized on the perinuclear location associated with the cytoplasmic side of the nuclear pore complex (NPC)—a complex that regulates the import and export of proteins and mediates global gene expression in cell models [75, 76]. RanBP2 also enhances the SUMOylation of HDAC4 and HNRNPM proteins, which are involved in mRNA splicing and transport [77, 78]. Although localization of Rhes on the NPC is not currently documented, we found that striatal Rhes coimmunoprecipitates with RanBP2 and Sec13 [9], two known components of the NPC. Rhes has also shown interactions with NPC-associated proteins, including HNRNP (L1 and H2 isoforms), during motor stimulation in the striatum [9]. Thus, we predict that Rhes may associate with the NPC to regulate the SUMOylation of targets involved in gene regulation, as well as mRNA splicing, another process shown to require SUMOylation [79]. In addition, Rhes may alter SUMO-independent signaling, perhaps by modulating mTOR and PKA signaling pathways that are linked to gene expression [7, 10, 80–82].

Aberrant SUMOylation has been linked to a variety of diseases, including cancer and neurodegenerative diseases. Blocking Rhes-mediated mHTT SUMOylation or the Rhes-E2 enzyme Ubc9 may therefore provide protection against HD [12, 25, 83]. Recent research has shown that HD is associated with significant changes in the nuclear envelope shape, aberrant localization of Ran guanosine triphosphatase (GTPase)-activating protein (RanGAP1), and improper nuclear export [84–86]. Since Rhes boosts the SUMOylation of RanGAP1 and binds to its SUMO E3 ligase, RanBP2, we anticipate that interfering with Rhes SUMOylation activities will likely benefit HD. Furthermore, the RanBP2/RanGAP1/Ubc9 SUMO E3 ligase pathway functions as a disassembler for nuclear pore export complexes [87]. Thus, Rhes, together with the RanBP2/RanGAP1/Ubc9 complexes, may aberrantly influence nuclear export pathways in HD, thereby acting as an attractive target for therapeutic interference.

Our new findings identify potential regulators of Rhes-mediated TNT-like membranous communication [19, 20]. We predict that Rhes may mediate the formation of TNT-like protrusions and the cell-to-cell transport of cargoes by a SUMO-dependent regulation of the expression of transcription factors, such as Mecf2, Egr2, Bhlhe22, which are known regulators of neuronal morphology and differentiation. Consistent with this notion, depletion of SUMO diminishes both the formation of TNT-like protrusions [19] and cell-to-cell transport, while also altering the Rhes-mediated expression of Mef2c and Bhlhe22 (Fig. 5).

Collectively, our results, obtained by a combination of high throughput interactomics, SUMO proteomics, gene expression analysis, and biochemical tools, demonstrate that Rhes promotes the SUMOylation of nuclear substrates involved in gene expression. Thus, Rhes may impact striatal function and dysfunction associated with neurological and neurodegenerative diseases via the SUMO-mediated regulation of gene expression.

### Supplementary Information

Below is the link to the electronic supplementary material.

Data file S1 (XLSX 77 KB)

Data file S2 (XLSX 25 KB)

Data file S3 (XLSX 138 KB)

Data file S4 (XLSX 26 KB)

Data file S5 (XLSX 8061 KB)

Data file S6 (XLSX 14 KB)

Supplementary figure 1 (TIF 507 KB)

Supplementary  figure 2 (TIF 775 KB)

Supplementary figure 3 (TIF 3696 KB)

Supplementary figure 4 (TIF 13131 KB)

## Data Availability

Enquiries about data availability should be directed to the authors.
