# Supplementary figures and images for: Rhes, a striatal enriched protein, regulates post-translational small-ubiquitin-like-modifier (SUMO) modification of nuclear proteins and alters gene expression
Source: Cell Mol Life Sci. 2024 Apr 8;81(1):169. doi: 10.1007/s00018-024-05181-8 (PMC11001699; doi:10.1007/s00018-024-05181-8)

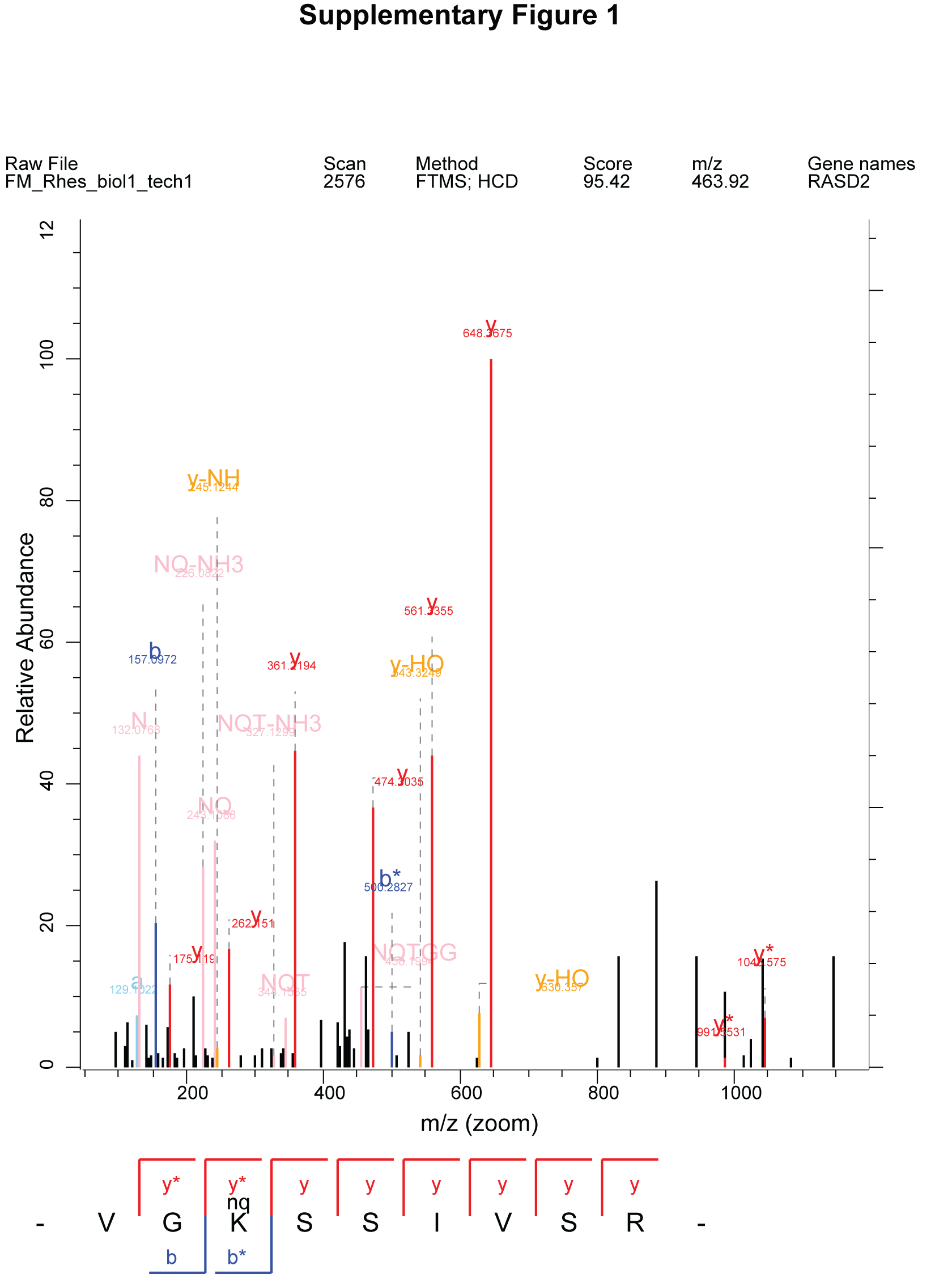

Supplement: Supplementary file 7 — Supplementary figure 1 (TIF 507 KB) [file 18_2024_5181_MOESM7_ESM.tif]

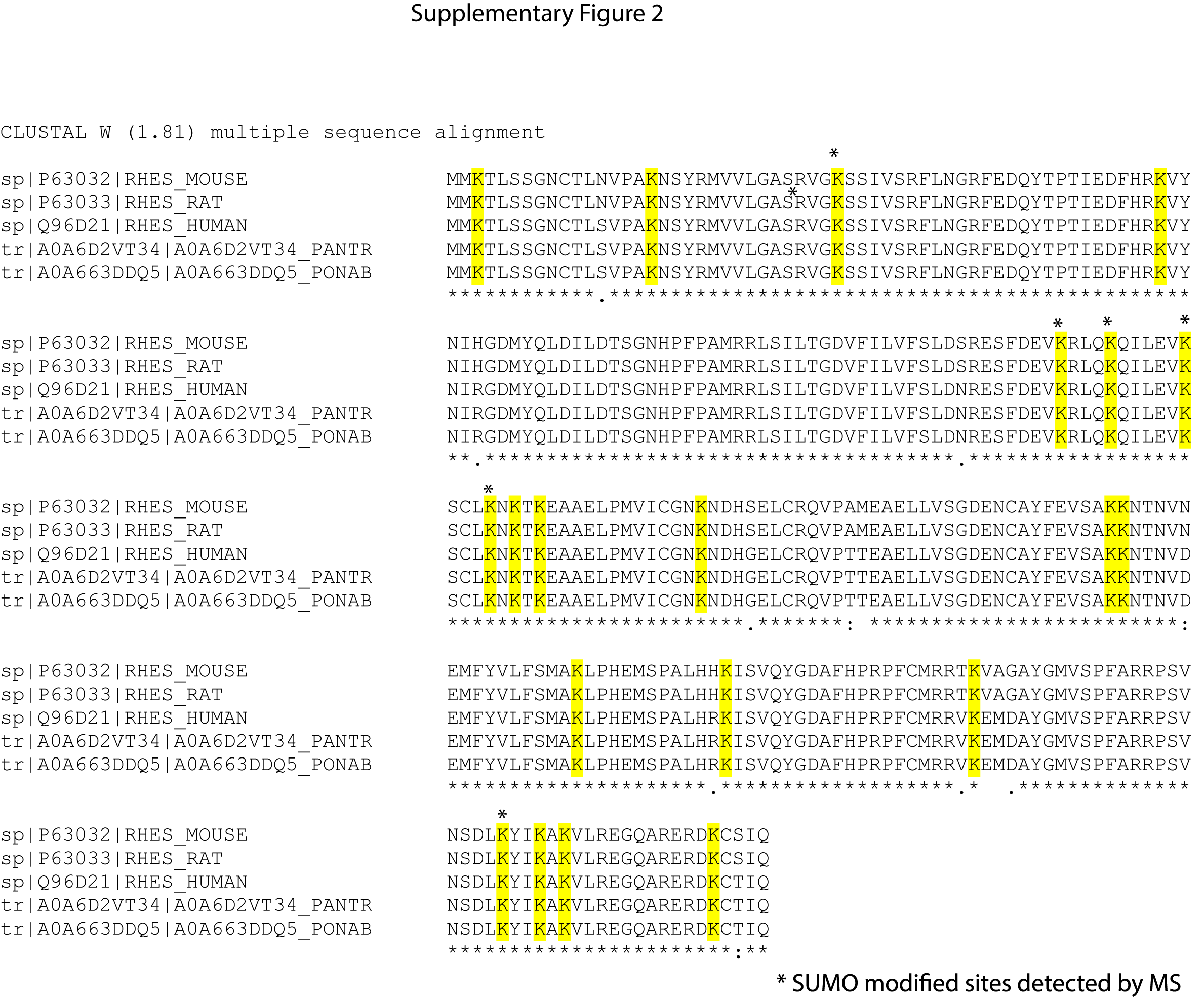

Supplement: Supplementary file 8 — Supplementary figure 2 (TIF 775 KB) [file 18_2024_5181_MOESM8_ESM.tif]

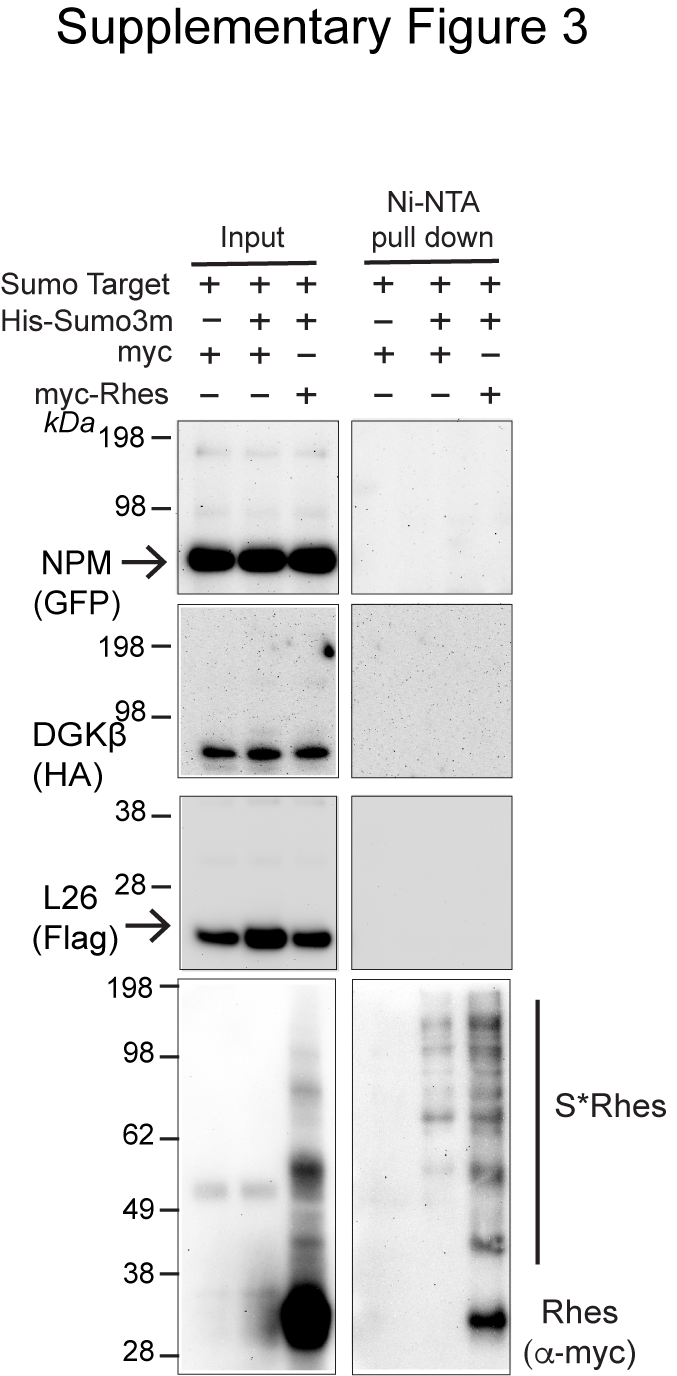

Supplement: Supplementary file 9 — Supplementary figure 3 (TIF 3696 KB) [file 18_2024_5181_MOESM9_ESM.tif]

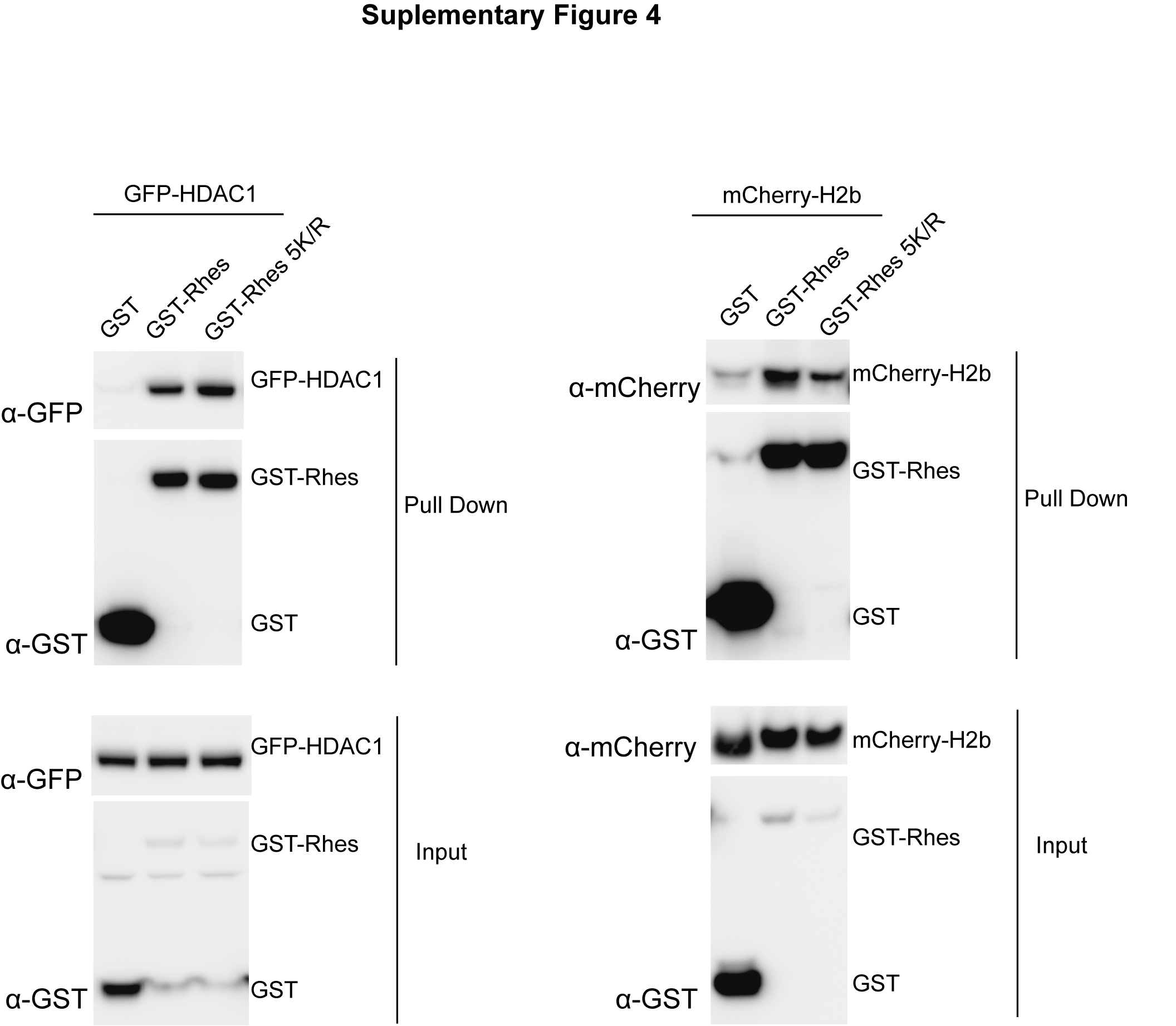

Supplement: Supplementary file 10 — Supplementary figure 4 (TIF 13131 KB) [file 18_2024_5181_MOESM10_ESM.tif]
